# Addressing Lameness in Farmed Animals: An Urgent Need to Achieve Compliance with EU Animal Welfare Law

**DOI:** 10.3390/ani9080576

**Published:** 2019-08-19

**Authors:** Elena Nalon, Peter Stevenson

**Affiliations:** 1 Eurogroup for Animals, rue Ducale 29, 1000 Brussels, Belgium; 2 Compassion in World Farming, River Court, Mill Lane, Godalming, Surrey GU7 1EZ, UK

**Keywords:** pain, animal welfare, cattle, pigs, chickens, sheep, goats, policy, legislation, European Union

## Abstract

**Simple Summary:**

In this short paper we explain the reasons why preventing and treating lameness in farmed animals can and should be considered a legal requirement under European Union (EU) animal welfare law. We also briefly present the situation in different farming sectors. We make the case that, in order to comply with current EU farmed animal welfare law, lameness prevalence and severity should be regularly monitored on farm, and species-specific alarm thresholds should be used to trigger corrective actions.

**Abstract:**

Lameness is the clinical manifestation of a range of painful locomotory conditions affecting many species of farmed animals. Although these conditions have serious consequences for animal welfare, productivity, and longevity, the prevention and treatment of lameness continue to receive insufficient attention in most farming sectors across the European Union (EU). In this paper, we outline the legislative framework that regulates the handling of lameness and other painful conditions in farmed animals in the EU. We briefly outline the current situation in different livestock farming sectors. Finally, we make the case for the introduction of regular on-farm monitoring of lameness and for the setting of alarm thresholds that should trigger corrective actions.

## 1. Introduction: The Legislative Framework

Whilst there are no species-specific EU welfare Directives for dairy cows, beef cattle, or small ruminants, this does not mean that these animals have no legal protection. They are covered by Directive 98/58 on the protection of animals kept for farming purposes (often known as the “General Farm Animals Directive”). Article 3 provides that farmers must “take all reasonable steps to ensure the welfare of animals under their care and to ensure that those animals are not caused any unnecessary pain, suffering or injury”. Although written in broad terms, this is a legally demanding provision requiring the taking of “all” reasonable steps to “ensure” welfare and the avoidance of unnecessary pain, suffering, or injury.

The European Commission has said—correctly, in our view—that to interpret this provision, one must look at the science and in particular at the reports produced by the European Food Safety Authority (EFSA) [1]. The science identifies what are the main welfare problems for each species that must be addressed and what are the “all reasonable steps” that must be taken to tackle them. Farmers with high levels of lameness who do not take all reasonable steps to reduce them are failing to comply with Article 3.

Pigs and chickens are covered by species-specific Directives, but the General Farm Animals Directive also applies to them. Accordingly, pig and chicken farmers must also take all reasonable steps to achieve low levels of lameness.

## 2. Lameness Is an Animal Welfare Problem in Most Livestock Farming Sectors

Addressing lameness is important from a legal perspective as its underlying conditions are painful and therefore require treatment. Additionally, lameness has a direct economic impact on farmers’ income as it compromises productive performances and can substantially decrease animal longevity.

Lameness is widely recognized as one of the major welfare problems for dairy cows as regards prevalence, duration and magnitude of adverse effect [2,3]. The EFSA states that the majority of estimates of lameness are within the range of 20% to 25% [4]. A recent study in the UK found that mean within-farm lameness prevalence was 31.6% [5]. There is an increased risk of lameness in intensive systems in which cows are housed throughout the year and/or produce high milk yields [2,6,7]. A report we prepared, based in part on a questionnaire sent to all Member States, shows that very few set alarm thresholds for the prevalence of lameness which, if exceeded, must be reduced by corrective action [8]. Although arguably a wealth of information is available from research, industry guidance, veterinarians, and farm assurance schemes, the persisting high prevalence of dairy cattle lameness indicates that prevention, detection, and treatment are still largely insufficient [9]. This is in part due to cultural and motivational factors [10,11]. However, farmers would benefit economically from reducing lameness as it is costly in terms of reduced milk production, potential shortened productive life, and treatment costs.

Fattening pigs and breeding sows are frequently affected by lameness and other locomotory disorders [12,13]. The reported prevalence for the EU is up to 20% for fattening pigs [12] and between 8.8% and 24.4% for breeding sows [14,15]. Apart from being an animal welfare problem, locomotory disorders are among the main reasons for early culling of otherwise healthy breeding sows [16]. There are several validated locomotion scoring scales [17,18], but the degree to which they are used in practice outside farm assurance schemes has not been thoroughly investigated. Farmers may find it difficult to decide when lame pigs need to be treated for pain and/or may not have familiarity with the use of anti-inflammatory drugs [19]. Additionally, information on preventive and corrective strategies for pig lameness may not always be readily available to farmers.

Sheep farming systems vary from extensive to intensive (or mixed). Lameness occurs in all systems and has been classified by EFSA among the three major animal welfare challenges for sheep [20]. The current estimated prevalence of sheep lameness varies from 5% to 10.6% in the UK [21,22,23]. Farmers tend to underestimate the proportion of lame sheep in their herds, and the decision to treat or not depends on various factors, including attitudes and beliefs, personality traits, and emotional aspects (e.g., sense of hopelessness, frustration, or lack of control) [22,23]. For these reasons, sheep lameness is most probably still under-detected and under-treated. Goat lameness is also emerging as an issue, particularly in dairy herds on high-concentrate diets [24] and as a consequence of infections [25].

Due to genetic selection for fast growth, the broiler chickens used in today’s intensive sector reach their slaughter weight three to four times as quickly as in the 1950s [26]. Owing to this genetically determined fast growth rate, many chickens suffer from painful leg disorders as their skeletal growth rate does not keep up with muscle growth rate. A large-scale UK study into leg disorders in broilers in 2008 found that 27.6% of the chickens had gait scores of 3 or more, i.e., lameness that is likely to be painful [27,28]. A 2016 report by the European Commission pointed out that around 30% of commercial intensively reared broilers presented leg abnormalities [26]. The use of fast-growing broiler genotypes that are vulnerable to high levels of lameness is arguably in breach of paragraph 21 of the Annex to Directive 98/58 which provides that “No animal shall be kept for farming purposes unless it can reasonably be expected, on the basis of its genotype or phenotype, that it can be kept without detrimental effect on its health or welfare”.

In parts of the EU, beef cattle farming is characterized by indoor confinement on hard flooring during the fattening phase, with energy-dense diets that are high in maize. Coupled with the genetic selection for rapid weight gain, these conditions predispose the animals to develop metabolic and joint disorders that can result in lameness [29], sometimes with extremely painful complications requiring the emergency slaughter of the affected animals [30]. There is limited information about the ability and willingness of farmers to address lameness in beef cattle, and preliminary studies suggest that much will depend on a more diffuse awareness of the advantages of preventing and treating its occurrence [31]. This is most certainly an area where further data collection is urgently needed as beef farming intensifies across the EU, with the increased risks associated with indoor confinement on hard flooring and/or lack of access to pasture (feedlot-style operations).

## 3. Discussion and Conclusions

Due to several concurring factors, lameness has become an important production disease in livestock farming, affecting many terrestrial farmed animal species. If left untreated, lameness can compromise animal health and welfare and can even lead to premature culling or death of the animals. To comply with EU law on the protection of animals kept for farming purposes, farmers must take “all reasonable steps to ensure the welfare of animals under their care and to ensure that those animals are not caused any unnecessary pain, suffering or injury”. The taking of effective steps to prevent lameness and the timely treatment of animals presenting signs of lameness are certainly part of this legal obligation.

Various strategies could be foreseen to encourage the prevention and treatment of lameness. Validated locomotion scoring systems have been developed for all the main farmed species [32] with guidance and training available for farmers and assessors. These systems could be easily integrated into existing official or voluntary controls. For instance, Germany included compulsory scoring of lameness as part of a set of animal-based indicators that pig farmers must use for self-assessment purposes [33]. Sheep farmers in the UK indicated that they would be favorable to a *bonus-malus* system whereby a prevalence of lameness above a certain threshold (≥10%) is penalized and a low prevalence (≤2%) is given economic incentives [22]. Plans to improve recognition and treatment of lameness in dairy cows have been developed by several private quality and assurance schemes [34,35], and a similar strategy could be adopted by national competent authorities. Monitoring systems whereby certain thresholds of lameness (e.g., >10%) trigger feedback to the farmer for corrective and follow-up actions appear to be particularly useful. In the case of broiler chickens, the use of slower-growing breeds with better animal welfare outcomes can be a strategy to reduce the current high incidence of painful leg disorders.

With a view to stimulating a more proactive attitude towards preventing and treating lameness, the industry and Member States should adopt a range of strategies tailored to the different farming sectors. The European Commission should produce a formal Recommendation (as they have done for the prevention of routine tail docking in pigs [36]) advising on the steps farmers must take to prevent and treat lameness as part of their legal duty to take all reasonable steps to ensure welfare and the avoidance of unnecessary pain, suffering, or injury.
